# Impact of Intergenerational Service Learning to Support Goal Attainment for Homebound Older Adults

**DOI:** 10.1093/geroni/igaf122.4136

**Published:** 2025-12-31

**Authors:** Yeonjae Lee, Meti Negassa, Maggie Ratnayake, Keith Chan

**Affiliations:** Johns Hopkins University, Baltimore, Maryland, United States; N/A, Pasadena, Maryland, United States; Lori’s Hands, Newark, Delaware, United States; Hunter College, New York, New York, United States

## Abstract

More than two million of older adults are homebound and five million need help leaving their homes. They often experience social isolation, food insecurity, and lack of connection to community resources. To date, home-based services for those aging in place are lacking. Using newly available data, this study examined the benefits of an intergenerational home-based service learning program on goal attainment in 1) social support, 2) home safety and cleanliness, 3) access to care resources, 4) food access, and 5) improving physicality for a community-based sample of 164 homebound older adults. Findings indicated that 75% reported positive changes in at least one of the five target service areas. The majority of participants reported 1) social support and 2) home safety and cleanliness as their most important goals. Results from chi square tests indicated statistically significant differences in goal attainment for 1) social support (Chi-square = 59.28, p < 0.001, Cramer’s V: 0.30) and 2) home safety and cleanliness (Chi-square: 29.50, p < 0.05, Cramer’s V: 0.21). Results from this study suggest that intergenerational in-home support services can improve social support and home safety and cleanliness for homebound older adults, thereby supporting aging in place. Policies and practice can support a pipeline of geriatric health professionals through innovative service learning models to benefit older adults, caregivers, and students.

